# Association Test Based on SNP Set: Logistic Kernel Machine Based Test vs. Principal Component Analysis

**DOI:** 10.1371/journal.pone.0044978

**Published:** 2012-09-13

**Authors:** Yang Zhao, Feng Chen, Rihong Zhai, Xihong Lin, Nancy Diao, David C. Christiani

**Affiliations:** 1 Department of Environmental Health, Harvard School of Public Health, Harvard University, Boston, Massachusetts, United States of America; 2 Department of Epidemiology and Biostatistics, School of Public Health, Nanjing Medical University, Nanjing, Jiangsu, China; 3 Department of Biostatistics, Harvard School of Public Health, Harvard University, Boston, Massachusetts, United States of America; Queen’s University Belfast, United Kingdom

## Abstract

GWAS has facilitated greatly the discovery of risk SNPs associated with complex diseases. Traditional methods analyze SNP individually and are limited by low power and reproducibility since correction for multiple comparisons is necessary. Several methods have been proposed based on grouping SNPs into SNP sets using biological knowledge and/or genomic features. In this article, we compare the linear kernel machine based test (LKM) and principal components analysis based approach (PCA) using simulated datasets under the scenarios of 0 to 3 causal SNPs, as well as simple and complex linkage disequilibrium (LD) structures of the simulated regions. Our simulation study demonstrates that both LKM and PCA can control the type I error at the significance level of 0.05. If the causal SNP is in strong LD with the genotyped SNPs, both the PCA with a small number of principal components (PCs) and the LKM with kernel of linear or identical-by-state function are valid tests. However, if the LD structure is complex, such as several LD blocks in the SNP set, or when the causal SNP is not in the LD block in which most of the genotyped SNPs reside, more PCs should be included to capture the information of the causal SNP. Simulation studies also demonstrate the ability of LKM and PCA to combine information from multiple causal SNPs and to provide increased power over individual SNP analysis. We also apply LKM and PCA to analyze two SNP sets extracted from an actual GWAS dataset on non-small cell lung cancer.

## Introduction

Rapid progress in high throughput genotyping technology has facilitated greatly the discovery of risk single-nucleotide polymorphisms (SNPs) associated with complex disease [1], [2]. At present, the population-based case control study is one of the most commonly used designs in genome-wide association studies (GWAS), with millions of SNPs being genotyped simultaneously from more than one thousand cases and controls. A standard approach to analyze GWAS data is to regress the phenotype on each genotyped SNP. However, due to the large number of SNPs, correction for multiple comparisons is necessary. As an example, for a GWAS with 1M SNPs, each SNP should be tested at the level of 5e-8 to maintain the overall α level at 0.05 [3], which may be too stringent to reach.

**Table 1:** Parameter settings of all simulations.

the simulated SNP Set	Scenario	Number of causal SNPs	Locations of the causal SNPs	Designed RR
CLPTM1L(31 SNPs)	A1	0	–	1.0
5p13.33	A2	1	1 of 31 in turn	1.1
	A3	1	1 of 31 in turn	1.2
	A4	2	15 and 22	1.1
	A5	2	11 and 22	1.1
	A6	2	7 and 14	1.1
	A7	2	11 and 30	1.1
	A8	2	6 and 29	1.1
	A9	2	11 and 26	1.1
	A10	2	11 and 29	1.1
	A11	3	All three-SNP combinations of 6,7,11,14,15,22,26,29,30	1.1
ASAH1 (154 SNPs)	B1	0	–	1.0
8p22	B2	1	1 of 154 in turn	1.2
	B3	2	26 and 81	1.1
	B4	2	81 and 126	1.1

It is possible that joint tests of multiple SNPs in linkage disequilibrium (LD) are more powerful than testing each SNP individually. There are several reasons for the superiority. Firstly, the number of tests is reduced if SNPs are tested by group rather than individually. Secondly, as the true causal SNP may not be genotyped directly, combining the information from multiple genotyped SNPs in LD with the causal SNP may be more effective than testing each SNP individually [4]. Meanwhile, a joint test can also examine whether a batch of biologically important SNPs are associated with the phenotype.

Several SNP set analysis methods have been proposed. The simplest way to determine the *p*-value of a SNP set is the individual SNP analysis. This method uses the smallest *p*-value of all SNPs, corrected for the number of effective tests, as the *p*-value for the entire SNP set [3]. The number of effective tests can be determined based on an upper bound for the overall type I error probability and pairwise correlations among SNPs. However, this method may not be optimal as it does not make full use of the LD structure among the genotyped SNPs. Gauderman et al. proposed a principal components analysis based approach (PCA), by which principal components (PCs) are computed from the SNP set and included in the regression model to test for the association [5]. Wu et al. proposed a powerful logistic kernel machine based test (LKM) to examine the association between the disease outcome and a SNP set [6]. Their simulations indicated that LKM has greater power than individual SNP analysis. On the basis of LKM, Wu et al. further proposed the sequence kernel association test (SKAT) which can test for the association between common or rare genetic variations in a region and a disease outcome [7].

In this article, we compare the performance of LKM and PCA by using simulated datasets. The structure of this article is as follows. The procedures of LKM and PCA will be briefly described. Results of several simulation studies are provided to compare test power and type I error rate between the two methods. We then demonstrate the application of these two methods to two SNP sets extracted from a Lung Cancer GWAS dataset, and finally discuss the implications of our results.

**Table 2:** Test power at the significant level of 0.05 for LKM and PCA in scenarios A4–A10.

							LKM				PCA		
Scenario	The causal SNPs	Genotyped	MAF	Position	Median *R* ^2^ with the genotyped SNPs	Individual SNP analysis	Linear	IBS	Linear weighted	IBS weighted	80%	60%	40%
A4	rs401681	Yes	0.43	15	0.53	0.739	0.883	0.852	0.257	0.270	0.710	0.794	*0.892*
	rs31489	Yes	0.40	22	0.56								
A5	rs421629	No	0.43	11	0.53	0.752	0.885	0.859	0.255	0.257	0.714	0.800	*0.894*
	rs31489	Yes	0.40	22	0.56								
A6	rs6554759	No	0.14	7	0.07	0.286	0.223	0.238	*0.521*	0.487	0.343	0.330	0.205
	rs10073340	Yes	0.13	14	0.06								
A7	rs421629	No	0.43	11	0.53	0.270	*0.402*	0.372	0.081	0.076	0.336	0.337	0.331
	rs27061	Yes	0.44	30	0.10								
A8	rs4975616	Yes	0.42	6	0.48	0.153	*0.224*	0.220	0.095	0.105	0.172	0.182	0.214
	rs27063	No	0.48	29	0.02								
A9	rs421629	No	0.43	11	0.53	0.698	0.850	0.825	0.238	0.234	0.656	0.746	*0.864*
	rs37008	No	0.45	26	0.48								
A10	rs421629	No	0.43	11	0.53	0.150	*0.233*	0.218	0.118	0.113	0.157	0.179	0.222
	rs27063	No	0.45	29	0.02								

The highest empirical power in each scenario is highlighted by using italics font.

## Methods

For a SNP set includes *p* SNPs from *n* individuals, let 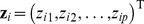 denote the *i*th individual’s genotypes. The disease outcome is denoted by *D*(1 = affected, 0 = unaffected).

### Logistic Kernel Machine Based Test

For the *i*th individual, LKM takes the form of
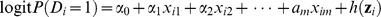



In the above equation, *α*_0_ is the intercept, *x_i_*_1_, *x_i_*_2_,…, *x_im_* denote *m* covariates to be adjusted for and *α*_1_ …, *α_m_* are their coefficients. The association between the SNP set and the outcome is modeled by a function *h*(.), which is defined by 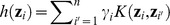 for some *γ*_1_, *γ*_2_,…, *γ_n_*. *K*(.,.) is a kernel function which measures the similarity between **z** *_i_* and **z** *_i_*_’_. Some choices for *K*(.,.) include the linear, identical-by-state (IBS), weighted linear and weighted IBS [6]. The linear kernel, 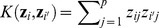, is the usual inner product between the vectors from 2 subjects. The IBS kernel measures the distance between individuals on the basis of the number of alleles shared identical by state (IBS) by a pair. Wu et al. suggested the use of the linear kernel if no epistatic effects are expected and the IBS otherwise. The weighted kernels impose weights to SNPs based on their allele frequencies or biological information.

Liu et al. described the connection between LKM and generalized linear mixed model (GLMM) [8]. They showed that *h*(.) could be regarded as a subject-specific random effect with a mean of zero and a variance of *τ* **K**. Thus a score test on *τ* = 0 could be used to test the null hypothesis of no association.

**Table 3:** Empirical type I error rates at the significant level of 0.05 for LKM and PCA.

				LKM				PCA			
Scenario	gene	α	Individual SNP Analysis	Linear	IBS	Linear weighted	IBS weighted	80%	60%	40%	20%
A1	CLPTM1L	0.05	*0.029*	0.050	0.048	0.052	0.051	0.050	0.051	0.050	0.050
		0.01	0.005	0.011	0.010	0.012	0.011	0.010	0.010	0.009	0.009
		0.001	0.000	0.000	0.001	0.001	0.001	0.001	0.001	0.001	0.001
B1	ASAH1	0.05	*0.026*	0.051	0.050	0.044	0.049	0.056	0.054	0.050	0.051
		0.01	0.006	0.010	0.011	0.008	0.010	0.012	0.011	0.011	0.009
		0.001	0.000	0.002	0.002	0.001	0.001	0.001	0.002	0.002	0.002

The type-I error rates significantly different from the nominal type-I error level are highlighted by using italics font.

### Principal Component Analysis Based Analysis

We first standardize each of the genotype by re-scaling it to have a mean of 0 and a standard deviation of 1. The variance-covariance matrix of the standardized SNP set is denoted as **V** *_p_*_×*p*_. Let 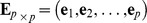 denote the *p p*-dimension eigenvectors of **V** *_p_*_×*p*_, and 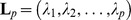 denotes the *p* corresponding eigenvalues, in which *λ*_1_>*λ*_2_>…> *λ_p_*. For the *i*th individual, the principal components are
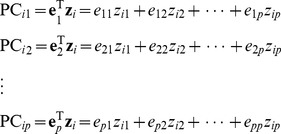



By definition, **e** *_l_* is selected to maximize the variance of PC*_l_* with the constraint that 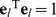. The covariance between PC*_l_* and PC*_l’_* is zero for *l*≠*l’*. *λ_l_* measures the variation explained by PC*_l_*. As SNPs in the set are always highly correlated with each other, the first few eigenvalues will be much greater than the others. This makes it possible to use the first few PCs to capture most of the variation in the SNP set. To do this, we only need to select the first *k* PCs with cumulative contribution 
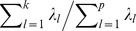
 greater than some threshold (eg. 80%). Instead of using the *p* SNPs, we will use the first *k* PCs in the multiple logistic model by:
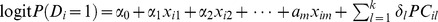



A *k*-df likelihood ratio test can be used to test the significance of the SNP set. For simplicity, we will use PCA(*Z*%) to denote the PCA with the PCs explaining at least *Z*% of the total variation. As example, if the top 2, 3 and 4 PCs explain 63%, 75% and 80% of the total variation, the corresponding *k*s for PCA(60%), PCA(70%) and PCA(80%) are 2, 3 and 4, respectively.

**Figure 1:**
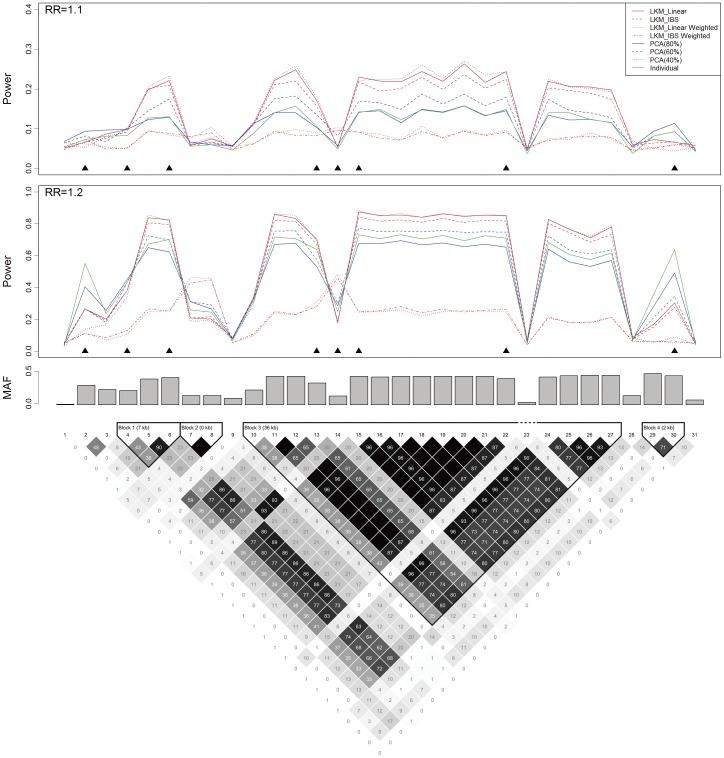
Power, the causal SNP and the genotyped SNPs in scenarios A2 and A3 based on the *CLPTM1L* gene. The top 2 plots show the power (*y*-axis) of each method over the locations (*x*-axis) of the causal SNPs. The triangles in the plot are the locations of the genotyped SNPs. The bar-plot shows the MAFs of all SNPs. The LD structure of the 31 SNPs is shown by the heat plot in the bottom of the figure, in which the gray scale indicates the value of *R* ^2^ (1 = black, 0 = white).

### Simulations

We use simulated datasets to evaluate the performances of LKM and PCA. Measurements for comparison include empirical type I error rate and test power. All the causal SNPs are assumed to improve the risk. We assume the disease model is
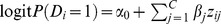



Here, *C* is the number of causal SNPs, *β_j_* is the log genetic relative risk (RR) of the *j*th causal SNP. We use a log-additive inheritance model in our simulation by imposing the assumption that risk multiplies with each additional allele. We let *C* = 0 to 3 in our simulations, denoting null, single causal SNP, two or three causal SNPs model.

**Figure 2:**
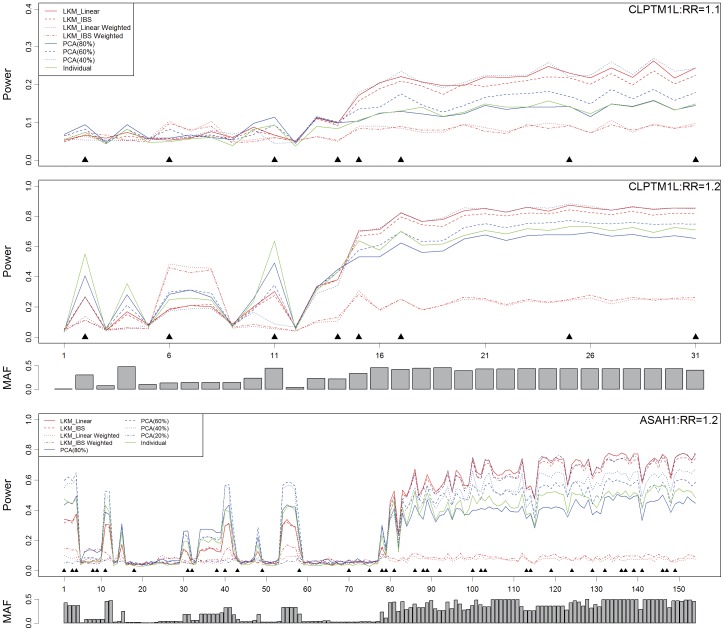
The relationship between the test power and the median *R* ^2^ between the causal SNP and the genotyped SNPs in scenarios A2, A3 and B2. The causal SNP on the *x*-axis has been ordered by the median *R* ^2^ in ascending. The test power of each causal SNP is plotted on the order of median *R* ^2^ between the corresponding SNP and the genotyped ones. The 2 bar plots in the figure represent the MAFs of SNPs in the *CLPTM1L* and *ASAH1* genes, respectively.

#### Simulations based on the *CLPTM1L* gene

Datasets are simulated on the basis of the *CLPTM1L* gene, a 27.35kb-long-gene located at 5p13.33. It encodes cleft lip and palate trans-membrane protein 1-like protein. Two SNPs in this gene, rs31489 and rs401681, were reported to be associated with non-small cell lung cancer (NSCLC) [9], [10]. We download the phased haplotypes of CEU (CEPH [Utah residents with ancestry from northern and western Europe]) samples from the website of the International HapMap Project (Phase 2, release 22). Thirty one SNPs, located within the range of 20kb upstream and downstream of the *CLPTM1L* gene, are used as the template of the simulated sequence. We use HAPGEN (version 2) to generate the simulated datasets [11], [12].

**Figure 3:**
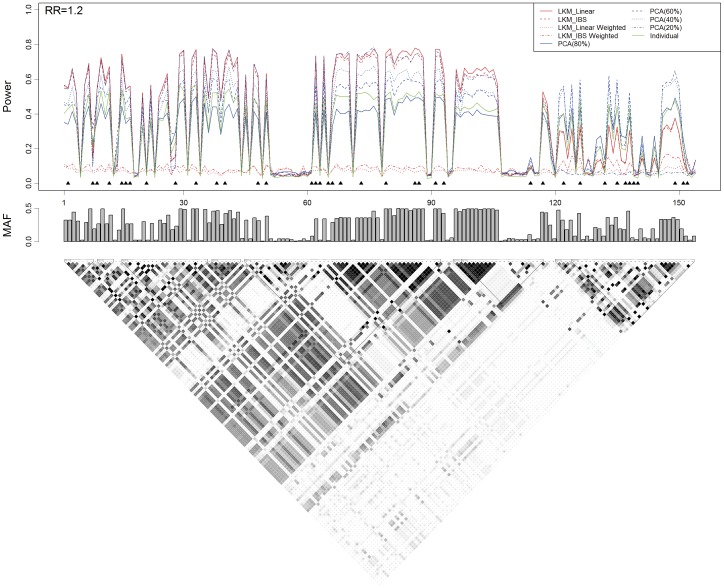
Power, the causal SNP and the genotyped SNPs in scenario B2 based on the *ASAH1* gene. The top plot shows the power (*y*-axis) of each method over the locations (*x*-axis) of the causal SNPs. The triangles in the plot are the locations of the genotyped SNPs. The bar-plot in the middle shows the MAFs of all SNPs. The bottom plot shows the LD structure of the 154 SNPs downloaded from the HapMap project.

We conduct 11 scenarios of simulations based on the *CLPTM1L* gene (scenarios A1–A11). Parameters of the simulations are described by Table 1. Scenario A1 is simulated to evaluate the performances of the two methods on controlling type I error. We generate 1,000 cases and 1,000 controls under the null disease model (*C* = 0), in which the outcome is independent of the loci. The empirical type I error rate is calculated as the proportion of rejecting the null hypothesis in the 5,000 simulated datasets. Scenarios A2 and A3 are simulated to compare the powers of LKM and PCA when there is only one causal SNP in the region. We set the genetic RR as 1.1 and 1.2 at scenarios A2 and A3, respectively. In both scenarios, each of the 31 SNPs in the *CLPTM1L* gene is set to be the causal SNP in turn. For each causal SNP, 1,000 datasets are simulated. The test power is calculated as the proportion of *p*-values less than 0.05. To make the simulations more realistic, only 8 of the 31 SNPs are used by LKM and PCA, which are directly genotyped by the Illumina 610k Quad chip.

**Table 4:** Test power at the significant level of 0.05 for LKM and PCA in scenarios B3–B4.

							LKM				PCA			
Scenario	The causal SNPs	Genotyped	MAF	Position	Median *R* ^2^ with the genotyped SNPs	Individual SNP analysis	Linear	IBS	Linear weighted	IBS weighted	80%	60%	40%	20%
B3	rs17126160	Yes	0.40	26	0.26	0.442	0.692	0.676	0.068	0.096	0.362	0.539	0.600	*0.715*
	rs6586684	No	0.39	81	0.40									
B4	rs6586684	No	0.39	81	0.40	0.088	0.199	0.192	0.050	0.056	0.149	0.206	*0.264*	0.158
	rs13263637	No	0.43	126	0.01									

The highest empirical power in each scenario is highlighted by using italics font.

We also examine the ability of these methods on utilizing information from multiple loci assuming that there are 2 or 3 causal SNPs with RR = 1.1 (scenarios A4 to A11). Both of the two causal SNPs are genotyped in scenario A4. In scenarios A5–A8, only one of the two causal SNPs is genotyped. No causal SNPs are genotyped in scenarios A9 and A10. Besides the number of genotyped causal SNPs, the difference among scenarios A4–A10 is reflected by the different median *R* ^2^ between the causal SNP and the genotyped SNPs. Details of these scenarios are presented in the first 6 columns of Table 2. In scenario A11, we assume that there are 3 causal SNPs. For each of the three-SNP combinations (84 in total) from the 9 causal SNPs used in scenarios A4–A10 (Table 1), 1,000 datasets are simulated.

**Table 5:** Results of LKM and PCA on the Analysis of the SNP sets from the Harvard Lung Cancer Susceptibility Study.

SNP set	Individual SNP Analysis		LKM				PCA			
	The least *p*-value in the SNP set	*p*-values for theSNP set	Linear	IBS	Linear weighted	IBSweighted	80%	60%	40%	20%
1	1.96E-6	1.23E-5	3.16E-6	6.96E-6	4.70E-2	1.01E-2	3.67E-5	2.56E-5	6.83E-6	6.83E-6
2	5.61E-6	6.74E-5	3.07E-4	1.78E-4	3.15E-1	1.44E-1	3.48E-6	8.67E-7	9.31E-1	9.31E-1

#### Simulations based on the *ASAH1* gene

*ASAH1*, acid ceramidase 1, is a 28.5-kb-long gene located at 8p22. It was reported to be associated with prostate cancer and Farber disease [13], [14]. The reference haplotype downloaded from HapMap includes 154 SNPs, with a more complex LD structure than the *CLPTM1L* gene. Four scenarios are simulated, in which scenario B1 evaluates the type I error and B2 to B4 evaluate the test power. In scenario B1, 5,000 datasets are simulated with no association between the disease outcome and the SNPs. In scenario B2, each of the 154 SNPs is set to be the causal SNP with a RR of 1.2 in turn. There are 1,000 simulated replicates for each of the causal SNP. Again, although the simulated datasets are generated using the overall 154 SNPs, only 39 genotyped SNPs are used in the analyses. Scenarios B3 and B4 both assume 2 causal SNPs with RR = 1.1. Both of the two causal SNPs are in strong LD with the genotyped loci in B3, while one causal SNP is in weak LD with the genotyped loci in B4. The causal SNPs in scenarios B3 and B4 are all common SNPs (MAF≈40%). The aim of these two scenarios is to evaluate the performance of LKM and PCA when causal SNPs are in the same or different LD blocks in a relatively large SNP set.

**Figure 4:**
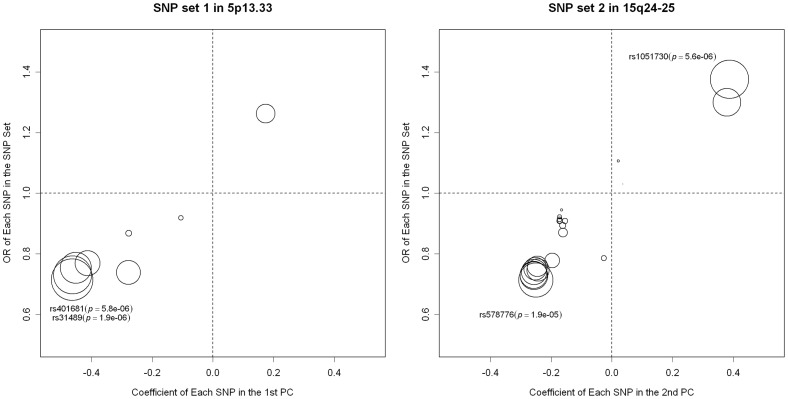
Loadings of the significant PCs on each of the SNP in the SNP sets from 5p13.33 and 15q24-25. The diameters of the circles in the plots are proportional to –log_10_(*p*-value).

### Application of LKM and PCA to two SNP Sets from a GWAS Dataset

We apply the two methods to test the significance of 2 regions extracted from a GWAS dataset studying the genetic susceptibility of non-small cell lung cancer (NSCLC). Details of participant recruitment for the study have been described previously [15]. This dataset includes 984 NSCLC cases and 970 controls recruited from Massachusetts General Hospital. DNA was extracted from the whole blood and genotyped using the Illumina 610k Quad chip. A total of 543,697 SNPs pass the general quality control (QC) procedure [16]. The first region includes 8 SNPs within a range including ±20kb of the *CLPTM1L* gene. The second one is an 116kb region, which includes 21 SNPs in 15q24-25. Genes in this region include the *CHRNA3*, *CHRNA5* and *CHRNB4*. These 3 genes were reported to be associated with nicotine addiction, smoking behavior and NSCLC [17]–[19]. Covariates adjusted in the analysis include age, gender(male/female) and smoking status (never/ever). The top 4 axes of variation generated by the EIGENSTRAT analysis are also included in the models to control for the confounding effect of population stratification [20], [21].

R package (version 2.13) is used to analyze both of the simulated and actual datasets. The *SKAT* package (version 0.73) is used to conduct LKM analysis. For the weighted kernel functions, we use the default weight of 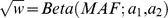, in which *MAF* is the SNP’s minor allele frequency, *a*_1_ = 1 and *a*_2_ = 25, *Beta*() is the density function of a *β* distribution. We also perform individual SNP analysis for each SNP set. To determine the *p*-value of a SNP set, a logistic regression model is fitted for each locus. The *p*-value of the set is then determined by the minimum *p*-value of all the loci, corrected by the effective number of tests [3].

## Results

### Empirical Type I Error Rate

The empirical type I error rate of LKM, PCA and individual SNP analysis are presented by Table 3. Both LKM and PCA control the type I error at the significant level of 0.05, 0.01 or 0.001. For the *CLPTM1L* gene, the mean numbers of PCs to explain at least 80%, 60%, 40% and 20% of the total variation are 3.00, 2.00, 1.00 and 1.00, respectively. For the *ASAH1* gene, the corresponding mean numbers of PCs are 6.85, 3.06, 1.92 and 1.00, respectively. The result indicates that type I error rate is independent of the number of PCs included in the model. The individual SNP analysis is conservative in both scenarios. Table S1 gives the standard errors of the type I error rate estimates.

### Empirical Test Power Based on the *CLPTM1L* Gene with Single Causal SNP

Results from scenarios A2 and A3 are presented by Figure 1. In general, both of LKM and PCA have power when the causal SNP is in high LD with the genotyped ones, which demonstrates their ability of “borrowing” information to increase the statistical power. Among the 4 LKM models, the one with linear kernel has the greatest power in most occasions. For PCA, we present the powers of PCA using PCs explaining at least 80%, 60% and 40% of the total variation, respectively. As the *CLPTM1L* gene is a small gene with a simple LD structure, only a very small number of PCs are needed to explain a large proportion (>40%) of the total variation. Thus the results from PCA(40%) and PCA(20%) are exactly the same. PCA(40%) is more powerful than the other PCAs when the causal SNP is at one of the 5^th^, 6^th^, 10^th^–13^th^ and 15^th^–28^th^ loci, as shown in Figure 1. Its power is very close to, or even greater than that of the LKM with linear kernel at some loci. This may due to the fact that when PCs in the model have already explained the difference between cases and controls, including more PCs will not improve the model, but instead exhaust more degrees of freedom and decrease the test power. However, if the causal SNP is in linkage equilibrium (LE) or is in weak LD with the genotyped SNPs, or located in a small LD block other than the one in which most of the genotyped SNPs reside in, including more PCs will improve the test power. This is confirmed by the causal SNP at one of the 1^st^–4^th^ and 29^th^–31^st^ loci, where PCA(80%) is dominant than LKM and the other PCAs. It is worth noting that although LKMs with linear or IBS weighted kernel do not have good performance in general, they are more powerful than the others when the MAF of the causal SNP is low (the 7^th^, 8^th^ or 14^th^ SNP in Figure 1).

The top 2 panels in Figure 2 illustrate the relationship between the test power and the LD between the causal SNP and the genotyped SNPs. As we expected, the test power for either of LKM and PCA increases as a function of the median *R* ^2^. As displayed in the left portion of this plot where the causal SNP is in LE or weak LD with the genotyped SNPs, PCA(80%) is more powerful than LKM and the other PCAs since more PCs are needed to “capture” the information that differentiates cases and controls. This is true except when the causal SNPs have low MAFs. It is not surprising that PCA(40%) and LKM with linear kernel have better performance than the others, shown at the right part of these 2 plots.

When compared to the individual SNP analysis, PCA and LKM are more powerful in most situations. However, when the causal SNP is in the 2^nd^, 29^th^ or 30^th^ locus with a RR of 1.2, the individual SNP analysis has greater power than the others. We believe this is not surprising because the inclusion of un-associated SNPs may “dilute” the effect of the causal.

### Empirical Test Power Based on the *ASAH1* Gene

Results of scenario B2 are shown by Figure 3. Because the LD structure of the *ASAH1* gene is more complex than that of the *CLPTM1L* gene, we also show the results from PCA(20%). The LD plot at the bottom of Figure 3 indicates that there are two LD blocks in the SNP set (the 1^st^ to the 119^th^ and the 120^th^ to the 154^th^). In the first block, the power from LKM with linear kernel and PCA(20%) is dominant than the others. Whereas in the second block, it is interesting to find that the power from PCA(60%) ≈ PCA(40%)>PCA(80%)>PCA(20%). PCA(60%) and PCA(40%) are also more powerful than LKMs. The influence of MAF, LD structure and number of PCs in the model on test power is also demonstrated by the bottom panel of Figure 2.

### Empirical Test Power Based on Genes with More than One Causal SNP

Results from scenarios A4 to A11 are presented by Tables 2 and S2. Once again, test power is affected by the strength of LD between the causal and genotyped SNPs. When both of the two causal SNPs are in strong LD with the genotyped ones (scenarios A4, A5 and A9), the power from LKM with linear or IBS kernel, as well as PCA(40%), is over 80%. The test power is dramatically reduced when at least one of the causal SNPs is in weak LD with the genotyped SNPs (scenarios A6–A8 and A10). When both of the causal SNPs are in weak LD with the genotyped SNPs (scenarios A6), PCA(80%) and PCA(60%) are more powerful than the other PCAs as a large number of PCs are necessary to capture the causal SNPs. For scenario A7, the powers generated from the 3 PCAs are very similar. Although PCA(80%) exhausts more degrees of freedom than PCA(40%), it may capture the information from the second causal SNP as a compensation by including more PCs. Due to the low MAFs, LKMs with weighted kernel functions are more superior to the other methods in scenario A6. The comparison of results from scenarios A4–A10 to those from A2, in which RR is also 1.1, demonstrates the advantage of LKM and PCA on combining information from several SNPs. Simulations based on the *ASAH1* gene from scenarios B3 and B4 generate similar results (Tables 4 and S3).

Scenario A11 in which there are 3 causal SNPs in the *CLPTM1L* gene yields similar conclusion that tests combing multiple SNPs tend to have higher power and the power increases with the strength of LD between causal and genotyped SNPs (Figure S1).

### Application on Harvard Lung Cancer GWAS

The results of the analysis are shown in Table 5. For SNP set 1, rs31489 from the *CLPTM1L* gene yields the least *p*-value of 1.96E-6 (1.23E-5 after the Bonferroni correction for the effective number of tests). The *p*-value of the LKM with linear kernel is 3.16E-6, the least of all LKMs. The least *p*-value of PCA (6.83E-6) happens when the PCs in the model explain 20% or 40% of the total variation. For SNP set 2, PCA (60%) is dominant than the other methods. LKMs with weighted kernels are less powerful than the other methods due to the fact that the possible associated SNPs are common ones (rs31489∶38.56%; rs1051730∶39.64%).

To understand how PCA utilizes the information from multiple SNPs, we also examine the coefficient of each SNP in the top PCs. For each SNP set, we regress the disease outcome on the top PCs. The 1^st^ PC from SNP set 1 and the 2^nd^ PC from SNP set 2, which are significant, are then presented by Figure 4. The significant PCs tend to have heavy loading on the “important” SNPs. As an example, for SNP set 1, the 1^st^ PC has heavy loadings on rs31489 (OR = 0.72, *p*-value = 1.96E-6) and rs401681 (OR = 0.73, *p*-value = 5.80E-6).

## Discussion

In this article, we compare the performance of logistic kernel machine based test and principal component analysis based test for the analysis of GWAS. Both of these two methods have the ability to test the association between a continuous or discrete outcome and a set of SNPs grouped by biological knowledge or genomic characteristics. We conduct extensive simulation studies using datasets generated based on the haplotypes downloaded from the International HapMap Project. We also apply these two methods to two SNP sets extracted from a GWAS data on NSCLC. The results demonstrate that both methods can correctly control the type I error. If the causal SNP(s) is/are in strong LD with the genotyped SNPs, both methods are superior to individual SNP analysis due to the ability of borrowing information from the genotyped SNPs in LD with the causal SNP(s). Furthermore, if there are two or more causal SNPs in a SNP set, LKM and PCA can combine their information and provide higher test power than individual SNP analysis.

Linear kernel performs well in most occasions in our simulation studies. Although slightly inferior to linear kernel, IBS kernel is claimed to have better performance when there is epistatic effect in the SNP set [6]. LKMs with weighted kernels are superior if the causal SNP has low MAF, as demonstrated by the results from scenario A6 (in which both causal SNPs have MAF <20%, as well as causal SNPs with low MAFs in scenarios A2 and A3 (the 7^th^, 8^th^ and 14^th^ SNPs).Thus LKM with weighted kernel may be a good choice when there is evidence that the MAF of the causal SNP is low. Meanwhile, when analyzing the same SNP set, if the weighted LKM generates a positive result while the un-weighted LKM does not, it is possible that the causal SNP in the region may have low MAF. Recent studies also indicate LKMs with weighted kernels has good performance when the disease is associated with rare variations [7]. The SKAT package in R also provides flexible weight specification options, which makes it possible to detect the association between disease and rare variations by giving the variations appropriate weights. This makes LKM a powerful and flexible tool in the coming age of next generation sequencing.

Test power from PCA is affected by the number of PCs included in the analysis. Simulation indicates that if the number of PCs is correctly specified, PCA may have even better performance than LKM. However, choosing the appropriate number of PCs in PCA may not be an easy task. In our simulation, we use PCA with different numbers of PCs to analyze SNP sets with simple or complex LD structure. For the simulation based on the *CLPTM1L* gene, if the causal SNP resides in the region in high LD with most of the genotyped SNPs, only the 1^st^ PC is needed to capture the information of the causal SNP. Including more PCs will decrease the power. While in the simulation based on the *ASAH1* gene, more PCs should be included in the model to increase the power if the causal SNP resides in the right side of the SNP set (as shown in the second LD block in Figure 3), in which only about 1/3 of the genotyped SNPs reside.

Thus it is critical to examine the LD structure of the SNP set before performing a LKM or PCA analysis. We suggest the SNP set to include SNPs in a LD block. In this situation, LKM and PCA with only a few top PCs may have good performance. However, if the SNP set covers a wide region containing several LD blocks, we suggest using PCA with PCs explaining a large proportion of variation to capture the information of the causal SNP(s). Chen et al. proposed a supervised PCA procedure for pathway-based analysis, in which only SNPs mostly associated with the disease outcome are used to extract PCs [22]. They used an approximate sampling distribution of the test statistics with a simulation-based standardization procedure to correct for the effect of pathway size. However, they used only the 1^st^ PC in the analysis, which has the risk of missing the causal SNP(s) if the LD structure of the SNP set is complex. A possible way to improve the power of PCA is to exclude the PCs independent of the causal SNPs. Although we can perform a feature selection procedure on PCs, such as only including significant PCs in the model, we risk increasing the type I error rate as the feature-selection procedure is ignored by the likelihood ratio test. A permutation procedure could be used to build the sampling distribution of the test statistic for the PCs after the feature-selection. However, this procedure is not efficient as one may need thousands of permutation samples to get a *p*-value small enough. More theoretical research should be taken for a solution to build a corresponding asymptotic distribution to improve the efficiency.

Both of LKM and PCA are capable of handling epistasis. PCA can also easily handle gene-environmental interactions, while it is still an issue for LKM. A possible limitation of PCA is the difficulty in interpretation of the results. However, just like LKM, a significant SNP set in PCA can be followed by a fine mapping or deep sequencing to identify the true causal SNP as the causal one should reside in the region in or close to the significant SNP set. Meanwhile, by checking the loading of the significant PCs on each SNP, we can identify which SNPs are more associated with the disease. By combining LKM or PCA with a moving window strategy, it is possible that we can have more precise information on where the causal SNP resides.

We acknowledge that our study has several limitations. Firstly, only LKM and PCA are evaluated, although there are several other SNP set based methods. In the LKM’s original paper [6], the comparison between LKM and other methods suggests that LKM is a powerful method. On the basis of their study, we further demonstrate that PCA, a traditional multivariate method, is comparable on many perspectives to LKM. Secondly, more complicated situations, such as rare variations and gene-gene interaction, are not included in the study. Further investigations are needed to address these issues.

## Supporting Information

Figure S1 **The relationship between the test power and the median** *R* **^2^ between the causal and the genotyped SNPs in scenario A11.** The *x*-axis in the top plot denotes the 84 three-SNP combinations, ordered in ascending by the median *R* ^2^ between the corresponding causal and the genotyped SNPs. The *y*-axis denotes the test power under each three-SNP combination. The bar plot in the bottom represents the median MAF of each combination.
(DOCX)

Table S1 **Standard error of the empirical type I error rate for LKM and PCA.**
(DOCX)

Table S2 **Standard error of the empirical power for LKM and PCA in scenarios A4–A10.**
(DOCX)

Table S3 **Standard error of the empirical power for LKM and PCA in scenarios B3–B4.**
(DOCX)
