# Supplementary material for: Association Test Based on SNP Set: Logistic Kernel Machine Based Test vs. Principal Component Analysis
Source: PLoS One. 2012 Sep 13;7(9):e44978. doi: 10.1371/journal.pone.0044978 (PMC3441747; doi:10.1371/journal.pone.0044978)

**Figure S1. The relationship between the test power and the median *R*^2^ between the causal and the genotyped SNPs in scenario A11.**

The *x*-axis in the top plot denotes the 84 three-SNP combinations, ordered in ascending by the median *R*^2^ between the corresponding causal and the genotyped SNPs. The *y*-axis denotes the test power under each three-SNP combination. The bar plot in the bottom represents the median MAF of each combination .


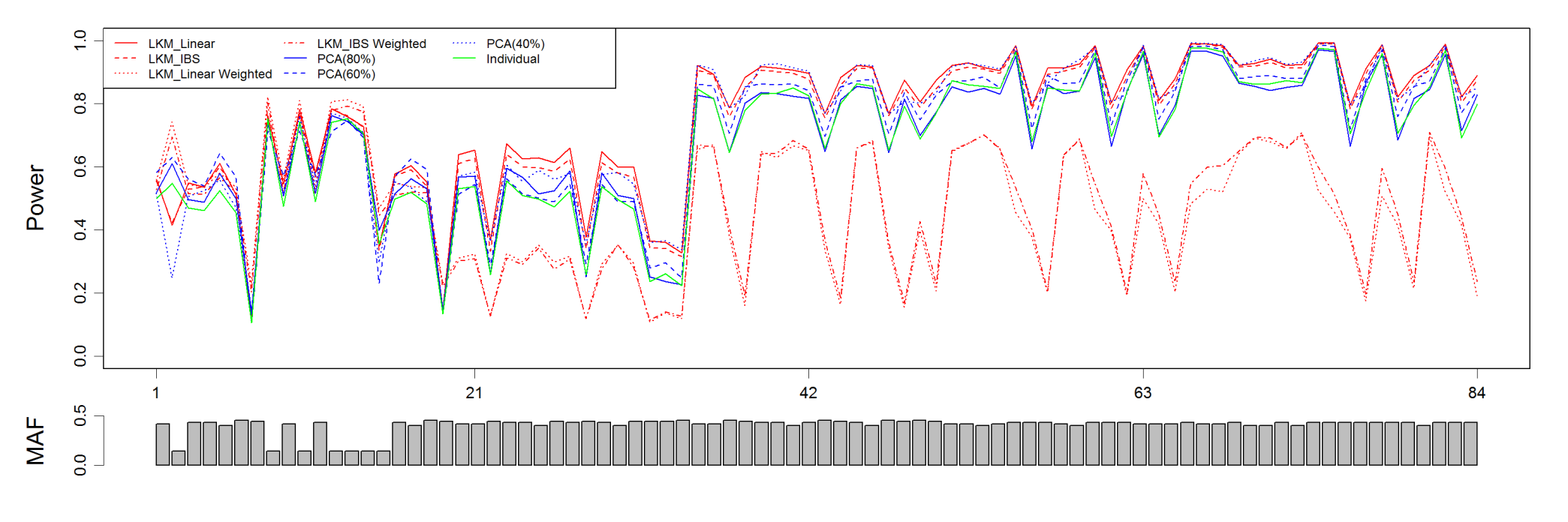

Supplement: Figure S1 — The relationship between the test power and the median R 2 between the causal and the genotyped SNPs in scenario A11. The x-axis in the top plot denotes the 84 three-SNP combinations, ordered in ascending by the median R 2 between the corresponding causal and the genotyped SNPs. The y-axis denotes the test power under each three-SNP combination. The bar plot in the bottom represents the median MAF of each combination. (DOCX) [file pone.0044978.s001.docx]
