# Supplementary material for: Association Test Based on SNP Set: Logistic Kernel Machine Based Test vs. Principal Component Analysis
Source: PLoS One. 2012 Sep 13;7(9):e44978. doi: 10.1371/journal.pone.0044978 (PMC3441747; doi:10.1371/journal.pone.0044978)
Supplement: Table S1 — Standard error of the empirical type I error rate for LKM and PCA. (DOCX) [file pone.0044978.s002.docx]

**Table S1. Standard error of the empirical type I error rate for LKM and PCA**

|  |  |  |  | LKM | | | | PCA | | | |
| --- | --- | --- | --- | --- | --- | --- | --- | --- | --- | --- | --- |
| Scenario | gene | α | Individual SNP Analysis | Linear | IBS | Linear weighted | IBS weighted | 80% | 60% | 40% | 20% |
| A1 | CLPTM1L | 0.05 | 0.0024 | 0.0031 | 0.003 | 0.0031 | 0.0031 | 0.0031 | 0.0031 | 0.0031 | 0.0031 |
|  |  | 0.01 | 0.0010 | 0.0015 | 0.0014 | 0.0015 | 0.0015 | 0.0014 | 0.0014 | 0.0013 | 0.0013 |
|  |  | 0.001 | 0.0000 | 0.0000 | 0.0004 | 0.0004 | 0.0004 | 0.0004 | 0.0004 | 0.0004 | 0.0004 |
| B1 | ASAH1 | 0.05 | 0.0023 | 0.0031 | 0.0031 | 0.0029 | 0.0031 | 0.0033 | 0.0032 | 0.0031 | 0.0031 |
|  |  | 0.01 | 0.0011 | 0.0014 | 0.0015 | 0.0013 | 0.0014 | 0.0015 | 0.0015 | 0.0015 | 0.0013 |
|  |  | 0.001 | 0.0000 | 0.0006 | 0.0006 | 0.0004 | 0.0004 | 0.0004 | 0.0006 | 0.0006 | 0.0006 |
