# Supplementary material for: Association Test Based on SNP Set: Logistic Kernel Machine Based Test vs. Principal Component Analysis
Source: PLoS One. 2012 Sep 13;7(9):e44978. doi: 10.1371/journal.pone.0044978 (PMC3441747; doi:10.1371/journal.pone.0044978)
Supplement: Table S2 — Standard error of the empirical power for LKM and PCA in scenarios A4–A10. (DOCX) [file pone.0044978.s003.docx]

**Table S2. Standard error of the empirical power for LKM and PCA in scenarios A4-A10.**

|  |  |  |  |  |  |  | LKM | | | | PCA | | |
| --- | --- | --- | --- | --- | --- | --- | --- | --- | --- | --- | --- | --- | --- |
| Scenario | The causal SNPs | Genotyped | MAF | Position | Median *R*^2^ with the genotyped SNPs | Individual SNP analysis | Linear | IBS | Linear weighted | IBS weighted | 80% | 60% | 40% |
| A4 | rs401681 | Yes | 0.43 | 15 | 0.53 | 0.0139 | 0.0102 | 0.0112 | 0.0138 | 0.014 | 0.0143 | 0.0128 | 0.0098 |
|  | rs31489 | Yes | 0.40 | 22 | 0.56 |  |  |  |  |  |  |  |  |
| A5 | rs421629 | No | 0.43 | 11 | 0.53 | 0.0137 | 0.0101 | 0.011 | 0.0138 | 0.0138 | 0.0143 | 0.0126 | 0.0097 |
|  | rs31489 | Yes | 0.40 | 22 | 0.56 |  |  |  |  |  |  |  |  |
| A6 | rs6554759 | No | 0.14 | 7 | 0.07 | 0.0143 | 0.0132 | 0.0135 | 0.0158 | 0.0158 | 0.0150 | 0.0149 | 0.0128 |
|  | rs10073340 | Yes | 0.13 | 14 | 0.06 |  |  |  |  |  |  |  |  |
| A7 | rs421629 | No | 0.43 | 11 | 0.53 | 0.0140 | 0.0155 | 0.0153 | 0.0086 | 0.0084 | 0.0149 | 0.0149 | 0.0149 |
|  | rs27061 | Yes | 0.44 | 30 | 0.10 |  |  |  |  |  |  |  |  |
| A8 | rs4975616 | Yes | 0.42 | 6 | 0.48 | 0.0114 | 0.0132 | 0.0131 | 0.0093 | 0.0097 | 0.0119 | 0.0122 | 0.0130 |
|  | rs27063 | No | 0.48 | 29 | 0.02 |  |  |  |  |  |  |  |  |
| A9 | rs421629 | No | 0.43 | 11 | 0.53 | 0.0145 | 0.0113 | 0.012 | 0.0135 | 0.0134 | 0.015 | 0.0138 | 0.0108 |
|  | rs37008 | No | 0.45 | 26 | 0.48 |  |  |  |  |  |  |  |  |
| A10 | rs421629 | No | 0.43 | 11 | 0.53 | 0.0113 | 0.0134 | 0.0131 | 0.0102 | 0.01 | 0.0115 | 0.0121 | 0.0131 |
|  | rs27063 | No | 0.45 | 29 | 0.02 |  |  |  |  |  |  |  |  |
