# Supplementary material for: Association Test Based on SNP Set: Logistic Kernel Machine Based Test vs. Principal Component Analysis
Source: PLoS One. 2012 Sep 13;7(9):e44978. doi: 10.1371/journal.pone.0044978 (PMC3441747; doi:10.1371/journal.pone.0044978)
Supplement: Table S3 — Standard error of the empirical power for LKM and PCA in scenarios B3–B4. (DOCX) [file pone.0044978.s004.docx]

**Table S3. Standard error of the empirical power for LKM and PCA in scenarios B3-B4.**

|  |  |  |  |  |  |  | LKM | | | | PCA | | | |
| --- | --- | --- | --- | --- | --- | --- | --- | --- | --- | --- | --- | --- | --- | --- |
| Scenario | The causal SNPs | Genotyped | MAF | Position | Median *R*^2^ with the genotyped SNPs | Individual SNP analysis | Linear | IBS | Linear weighted | IBS weighted | 80% | 60% | 40% | 20% |
| B3 | rs17126160 | Yes | 0.40 | 26 | 0.26 | 0.0157 | 0.0146 | 0.0148 | 0.0080 | 0.0093 | 0.0152 | 0.0158 | 0.0155 | 0.0143 |
|  | rs6586684 | No | 0.39 | 81 | 0.40 |  |  |  |  |  |  |  |  |  |
| B4 | rs6586684 | No | 0.39 | 81 | 0.40 | 0.0090 | 0.0126 | 0.0125 | 0.0069 | 0.0073 | 0.0113 | 0.0128 | 0.0139 | 0.0115 |
|  | rs13263637 | No | 0.43 | 126 | 0.01 |  |  |  |  |  |  |  |  |  |
